# Fostering Interprofessional Education and Practice: Organizational Outcomes of a Faculty Development Program for Educators Across Health Professions

**DOI:** 10.7759/cureus.85022

**Published:** 2025-05-29

**Authors:** Gayle Haischer-Rollo, Diane Hale, James D Honeycutt, Rhiana Saunders, William Bowers, Ryan Sanborn, Reece Tuckerman, John Nowell, Thomas McFate, Jessica Servey

**Affiliations:** 1 Department of Faculty Affairs and Faculty Development, Uniformed Services University of the Health Sciences, Bethesda, USA; 2 Department of Surgery, Brooke Army Medical Center, San Antonio, USA; 3 Department of Family Medicine, Mike O’Callaghan Military Medical Center, Clark County, USA; 4 Department of Obstetrics and Gynecology, Brooke Army Medical Center, San Antonio, USA; 5 F. Edward Hebert School of Medicine, Uniformed Services University of the Health Sciences, Bethesda, USA

**Keywords:** culture change, educational culture, faculty development, interprofessional education, organizational culture

## Abstract

Background

The role of clinician educators has expanded to encompass diverse responsibilities alongside providing competent care and engaging in scholarly activities. Faculty development (FD) programs have emerged as vital tools for equipping educators with teaching skills and career advancement opportunities. However, assessing faculty development programs’ impact on organizational culture remains challenging due to their multifaceted nature. This study explores this gap to understand how faculty development influences organizational culture.

Methods

This qualitative study used a constructivist approach to explore faculty perspectives on the Uniformed Services University of the Health Sciences (USU) Faculty Development Program’s influence on organizational culture. Forty-three faculty members participated in nine semi-structured focus groups, selected for diversity across roles and locations. Interviews were recorded, transcribed, and anonymized to ensure accuracy and participant confidentiality.

Data analysis

Transcripts were analyzed using thematic analysis. A multidisciplinary team independently reviewed transcripts, identified preliminary codes, and refined themes through iterative consensus building. Coding was organized using memos and grouped into themes. Using information power principles, the team determined that nine focus groups provided sufficient data richness to meet the study’s aims.

Results

The research team identified and refined four central themes: professionalization, legitimization, the value of community, and leadership impact. The participants reported a greater sense of professionalization through enhanced teaching skills, increased awareness of educational literature, and heightened confidence in their educator roles. The faculty development program fostered a strong community, promoting collaboration, knowledge sharing, and mutual support across specialties and locations. Leadership impact was evident at individual and institutional levels, increasing confidence, mentorship, and organizational support.

Conclusions

This study provides valuable insights into the organizational impact of faculty development programs, shedding light on how such initiatives influence faculty experiences and organizational culture. Future research should explore leadership values, faculty retention, burnout prevention, and the broader value of education within academic institutions.

## Introduction

Clinician educators’ skills and expectations continue to expand with the new roles such as role model, skills assessor, content creator, and leader. In addition, these educators face traditional expectations such as providing competent care, learning new technology, and engaging in scholarly activities [1-3]. Faculty development (FD) helps educators navigate these roles by equipping them with skills and knowledge for effective teaching, career advancement, and enhancing academic systems.

Assessing FD impact is complex, given its incremental influence on behavior and attitudes. While genuine behavior change arises from multiple factors, studies indicate that FD programs can enhance individual confidence in educational skills, improve observed skills, and increase academic promotion rates [1-9]. Furthermore, FD can facilitate leadership roles and strengthen interdepartmental and interorganizational networking [3,6,8,10-13]. Researchers employ diverse methods to evaluate FD’s impact to include attendee surveys, including pre- and post-assessments and retrospective comparisons [6,8,10,14,15], while others conduct participant interviews [6,7,11,15], analyze participant curriculum vitae [13,15], or employ control groups [11]. The evaluation of FD programs often focuses on Kirkpatrick’s levels 1 and 2 (satisfaction, knowledge, and skills), with fewer studies examining level 3 (behavior change) [1,8]. The few studies that examine level 3 assessments utilized peer [4,16], supervisor [16], and student evaluations [9] to quantify impact.

Faculty development has the potential to transform organizational culture and can be used intentionally for that purpose. Describing organizational culture is complex due to its intangible nature. As organizational members, clinician educators often embody culture through daily behaviors, obscuring its distinct features. Schein delineates three cultural levels [17]. The superficial level, or artifacts, includes observable aspects such as organizational charts and awards. The second level encompasses beliefs and values, guiding artifact interpretation and revealing interaction patterns, norms, and social validation. The third and most elusive level involves underlying assumptions, deeply ingrained beliefs shaping perceptions and power dynamics [17]. In faculty development, culture is reflected in the community of educators and their engagement with the institution. At the artifact level, this may include awards or certification for teaching excellence. At the level of beliefs and values, it involves how the teaching role is communicated and valued. At the level of underlying assumptions, it is reflected in structural elements such as the time protected for educational preparation, the integration of teaching into promotion criteria, and whether leadership explicitly prioritizes education as a part of professional growth.

While a few studies have explored the organizational impact of faculty development (FD), they often rely on individual interviews with a mix of academic and nonacademic stakeholders, including peers and supervisors, rather than broad-based faculty perspectives [16,18]. In some cases, organizational change is only implied, with measurement limited to individual-level outcomes [11]. For example, one case study noted institutional shifts in the perceived value and credibility of educators [9], and another described how FD participants were seen by peers and supervisors as contributing to a culture of lifelong learning [16]. However, these studies tend to focus on highly structured, longitudinal programs with small cohorts, which may limit the ability to capture broader cultural or institutional change. To understand the long-term impact of FD, it is essential to investigate shifts in the institutional value placed on teaching, as well as changes in structural support for educational roles [1]. Despite the growing recognition of organizational culture as a critical component of educational change, there is a lack of studies that directly examine cultural impact at scale. To our knowledge, no published studies have used focus groups to explore faculty-wide perspectives on how FD influences organizational culture. Our study addresses this gap by using focus groups to gather collective faculty insights, offering a novel approach to understanding the broader organizational effects of FD.

The FD program at the Uniformed Services University of the Health Sciences (USU) delivers and supports faculty development at our 22 core teaching hospitals located across the United States. It serves over 6,500 faculty appointed in the School of Medicine (SOM) and other faculty within additional university schools with an infrastructure located at our single SOM. The faculty attending are a mix of active-duty military, reservists, and civilian healthcare providers. The program was formally started in 2011, with an increase and categorization of standardized content in 2014 shared among a small group of experienced faculty developers [19]. To meet the needs of our large and dispersed faculty, the program was further expanded in 2017, adding an intentional training for faculty developers to deliver a predetermined set of standardized courses in a universal manner [19]. Currently, there are 40 trained faculty developers who deliver the iteratively updated set of content, with a dozen more experienced faculty developers sharing more advanced content, allowing greater accessibility for a larger number of faculty. Since 2020, the program has averaged approximately 600 workshops annually, with more than 1,800 unique individuals attending numerous workshops yearly. Of the participants, 70%-75% are physicians, and the remainder are from a myriad of health professions such as dentists, nurses, pharmacists, physician assistants, and basic scientists.

Understanding how faculty development influences organizational culture is critical for aligning educator experiences with institutional goals. By examining faculty perspectives through the lens of Schein’s three levels of organizational culture, this study offers insights that can inform future program design, foster cultural cohesion, and support systemic change.

Some of the data from this article were included in a manuscript that was submitted to a medical student research competition and was uploaded to a server for judging.

## Materials and methods

A constructivist approach was well-suited for this study, allowing for the in-depth exploration of faculty perspectives on how the USU Faculty Development Program influences organizational culture within the Military Health System (MHS). The study aimed to understand not only the perceived value of the program but also the factors that strengthen or undermine organizational culture.

Data collection

Data were collected using transcripts from focus group interviews of faculty members. Focus groups were chosen given the complexity of the data, the number of variables, and the inherent need for collaboration when investigating organizational change [20]. Before conducting the focus groups, WB, RS, JN, and RT underwent a 90-minute training session. The session included didactics on the purpose and principles of focus groups, tips and tricks for maximizing effectiveness, and a role-play session for practical application and feedback.

Interview process

The Faculty Development Program contacted learners (faculty members) who attended at least one workshop in the USU FD Program during both the calendar year period of 2014-2017 and after 2021 to participate in a focus group. These dates allowed for the inclusion of faculty based on their participation in the program both before and after its expansion, allowing for the direct comparison of the educational and organizational culture across these time periods. Learners included any health profession educator in our organization such as physicians, dentists, scientists, nurses, and other health professionals, who attended workshops at any of the teaching hospitals or the university. A “request to participate” email was sent by JS with instructions to contact TM if they were interested in participating. We formed focus groups by randomly selecting the participants based on availability, ensuring a diverse mix of specialties and locations. This approach intentionally captured a realistic cross-section of national faculty, fostering a broad range of perspectives. Each focus group was hosted by two members of the research team, which included JN, RS, RT, and WB (TM provided support for one meeting). One host acted as the primary interviewer, and the other provided support for recording the meeting, attendance tracking, monitoring the chat, and clarifying comments, as needed. They utilized a semi-structured interview guide developed by JS, GH, JH, DH, and RDS and utilized Schein’s model as a focusing lens (Appendices). The meetings were held and recorded using Google Meet (Google, Inc., Mountain View, CA). All participants used aliases during the interview to enhance the anonymity of the recorded transcripts. We chose interviewers who were medical students to avoid introducing bias or the perception of organizational change where it was not present. The remainder of the research team would be known to the faculty being interviewed and could have had an undue influence on the interviewees. Recordings of focus group interviews were transcribed by paid transcription services (Rev.com and Transcription Hub). The transcriptions of the recordings were reviewed by JN, RS, RT, and WB to ensure accuracy and the maintenance of anonymity. This study was reviewed and approved by the Uniformed Services University of the Health Sciences Institutional Review Board (IRB) (approval number: LDBS.2022.371).

Study population

A total of 3,806 individual learners were identified in the attendance data report, of which 568 attended faculty development sessions within both time periods and were invited to participate in the focus groups. Seventy-six individuals responded, of which 62 were scheduled to attend, and 43 were able to participate in one of the 10 focus groups scheduled between February 2, 2023, and March 13, 2023. The remaining 19 individuals were unable to meet secondary to scheduling conflicts. The maximum number of participants in a group was eight, and the minimum was two. The 43 participating faculty represented 15 different departments. This included physicians and dentists of distinct specialties and subspecialties and several scientists with different backgrounds and ranks and from 17 unique locations across our organization (Table 1). The participants had attended an average of 56.5 hours of FD (10.5-129.5 hours).

**Table 1:** Demographics of the Participants (N=43) GME, graduate medical education; USU, Uniformed Services University of the Health Sciences

Category	N
Female	21
Male	22
Adjunct associate professor	1
Assistant professor	20
Associate professor	15
Professor	5
Unknown academic rank	2
Department (based on primary appointment)	
Anesthesiology	3
Family medicine	7
Gynecologic surgery and obstetrics	7
Medicine	3
Military and emergency medicine	1
Neurology	1
Pathology	2
Postgraduate dental college	5
Pediatrics	8
Pharmacology and molecular therapeutics	1
Preventive medicine and biostatistics	2
Psychiatry	2
Surgery	1
Location size (based on number of programs)	
Large (10+ GME programs and USU)	27
Medium (3-10 GME programs)	12
Small (1-2 GME programs)	4

Data analysis

All the reviewed transcriptions were independently read through by the coders (JS, GH, JH, DH, TM, and RDS), who identified preliminary recurrent concepts, words, and themes. Consistent with thematic analysis, the team members then discussed several concepts identified from the focus group question responses [21,22]. Coding memos were created and shared among the group to capture concepts. From these memos, repeated ideas and perspectives were tagged with codes. In an initial meeting, the group debated and refined themes through a consensus-building process where individual perspectives and assumptions were discussed to enhance understanding. All coders then met to discuss all the transcriptions and repeated the consensus-building process. Through this iterative process, codes were grouped into categories that reflected emerging patterns in the data. This coding structure informed the subsequent inductive analysis and guided theme development. From there, the coders were divided into two groups (JS, DH, RDS, GH, JH, and TM), and each group coded five transcriptions and met separately to discuss coding and resolve any conflicts. Finally, the study team met, discussed the codes from all transcripts, and refined the codes into themes. One member (GH) reviewed the transcripts again, using the developed codes to assess their appropriateness, confirm their meanings, and identify any additional themes that may have been overlooked. Utilizing information power, the team decided that we had a narrow question, dense specificity, applied theory, and strong dialogue, and thus, nine focus groups were sufficient [22].

Reflexivity

JS is a family medicine physician who has served as an associate dean of faculty affairs and/or faculty development for the past 10 years. GH, a neonatologist, has participated in and now teaches faculty development sessions as an assistant dean of faculty development. DH is a general surgeon, surgery residency program director for two years, faculty member for eight years, and a Faculty Development Outreach Certification of the Uniformed Services (FOCUS) facilitator for four years and participant for eight. JH, a civilian family physician, has been a faculty member in a family medicine residency for 12 years and a FOCUS facilitator for five years and serves as a military reservist on the USU family medicine department faculty. RDS, a reproductive endocrinologist, is currently an associate dean of quality improvement and patient safety, a faculty member for 12 years, and a FOCUS facilitator for three years. TM, a civilian with a PhD in neuroscience, has developed and managed the USU Faculty Development Program since 2015. JN is a second-year medical student at USU and a naval officer with nine years of service, including experience in surface warfare and shipboard navigation training. RS is a second-year medical student with a background in clinical research in orthopedic surgery and sports medicine. WB, a third-year medical student and army officer of eight years, previously served as a combat engineer.

During the interpretation, coding, and consensus-building process, the research team openly discussed their individual experiences, perspectives, and potential biases related to their roles within the organization and their involvement in faculty development, as both participants and facilitators. These discussions informed a participatory approach to theme development [23].

## Results

The thematic analysis identified four themes from the coding discussions: professionalization, legitimization, the value of community, and the impact of leadership.

Professionalization of the clinician educator’s role

The idea of the professionalization of the clinician educator’s role was repeatedly described. This theme embodied the skills and transferability of skills to other areas of the faculty members’ lives. Many participants described how the FD program provided evidence-based teaching strategies, which enhanced their self-confidence and deepened their engagement with the medical education literature. This foundation not only improved their individual teaching practices but also empowered them to address educational challenges and implement meaningful changes in their clinical academic environments. Additionally, participation in the faculty development (FD) program played a pivotal role in shaping faculty members’ decisions to remain in academic medicine beyond their military service.

Faculty credited the FD program with helping them understand how their day-to-day work contributes to the broader mission of training interns and residents. Over time, this growing identification as educators represented more than personal growth; it marked the beginning of a cultural shift within their institutions. As more individuals embraced education as a skilled and valued component of their professional identity, their collective mindset influenced how education was perceived and supported at the organizational level (Table 2).

**Table 2:** Representative Quotes for Professionalization USU: Uniformed Services University of the Health Sciences

Professionalization Themes	Representative Quotes
Increase perception that teaching is a skill	I would say there’s been (increased) emphasis that teaching is a separate skill set…just having some sort of foundation and how to teach in developing that skillset is been critical (focus group 6).
Awareness of the body of literature and an evidence-based way to teach	I was unaware prior to the (USU) faculty development course there was literature out there on adult learners, and I’ve read a few of those papers and have used it to revamp part of our curriculum, and I think it’s making the fellows more engaged (focus group 9).
Change in the confidence of teaching skills	Because I think seeing yourself as an educator, I feel a lot about building confidence in your ability to help people achieve whatever learning objectives you’re trying to accomplish. So, I feel like that building confidence piece, I think that participating in faculty development helps with that a lot (focus group 3).

Enhancing academic legitimization and professional standing

Enhancing academic legitimization and professional standing signifies the process through faculty development by which the perceived validity and importance of academic roles are strengthened, leading to increased recognition and respect for faculty members within and beyond the organization. The focus groups’ comments highlighted the legitimization and credibility of being an academic faculty member within the organization. The participants described this through the increased recognition of the importance of academic advancement and a shift in the credibility, value, and respect given to academic faculty at the outlying hospitals not geographically close to the actual “university.” In our organization, this is noteworthy because teaching is not the singular identity of our healthcare organization. In fact, other aspects of healthcare such as battlefield medicine are often seemingly more valued in the larger organizational culture. To add to the legitimization in the culture, faculty who participated in FD would recruit other faculty to attend, garnering similar understanding and skillsets. An example discussed was the increased engagement of a previously hard-to-reach subgroup (surgery subspecialties) in FD sessions after observing surgeon-delivered topics. Through the interprofessional learning environment, the participants acknowledged that they grew their networks and gained interdepartmental credibility from other faculty participants. Specific subthemes identified were the impact of provided tools for change and advancement within the organization, the enhanced understanding of the importance of academic rank and advancement, recognition that skills were transferable within and outside of the military organization, and personally experienced increased credibility and the recognition of their academic role (Table 3). Multiple participants discussed the knowledge of the process of attaining academic promotion and the requirement of a certain academic rank with some academic leadership roles (e.g., department chair) that were provided through the FD program. Connecting to the larger academic culture in the United States added external validation and legitimization.

**Table 3:** Legitimization Representative Quotes USU, Uniformed Services University of the Health Sciences; FD, faculty development

Legitimization Themes	Representative Quotes
Advancement in the organization	And because of faculty development and its impact on my teaching academic career, I was able to achieve a faculty promotion of associate professor, non-prefixed. I accepted a job with USU as a clinician educator. So yeah, thankful for eight years of faculty development that helped vector me toward a career in education (focus group 6).
Importance of academic advancement	In the last five years, I would say I’ve observed an increase in participation in the, in pursuing academic advancement to associate professor level. A lot more faculty are appearing to do that than they did in the past (focus group 7).
Credibility of educators	I think the FD program gives the younger generation of teachers and leaders some sort of scientific basis, a central source of information without it seemingly coming from outer space or YouTube. It’s now a sanctioned source that is respected for information that really applies to medical, dental, whatever you’re teaching (focus group 5).

Value of community

The value of community refers to the shared sense of connection, collaboration, and identity that faculty experienced through participation in faculty development programs. This theme captures how common language, interprofessional networks, and collective learning fostered ongoing relationships, reduced isolation, and supported both professional growth and educator identity formation. Multiple participants voiced their appreciation for the development and value of community through this program. This appreciation was evident through the discussion of shared language, building commonalities, and increased collaboration. The participants discussed how local faculty developers encouraged and reinforced interdepartmental and interprofessional networks. As faculty changed locations and the program added virtual FD workshops, this community network was extended beyond the local institution, potentially including multiple hospitals across thousands of miles. The extended network was welcoming and collegial to all FD participants. These connections broke historic silos by creating a common language of education and shared experiences, which enabled faculty to share ideas on how to approach difficult clinical educator scenarios, collaborate on educator-relevant projects, and troubleshoot similar leadership challenges. One specific example mentioned numerous times by name was the “Learners in Trouble” workshop. It was described as a memorable and impactful educational experience that shaped teaching styles and approaches to struggling learners including discussing with faculty outside of their own program or department (which traditionally would not be done). Many groups also described experiencing a sense of community in education, which motivated faculty to attend future workshops and collaborate repeatedly with new colleagues and helped facilitate their identity formation as educators. Group identity formation emerged, and its members progressively invited more colleagues into the community. In sum, a snowball phenomenon developed; motivation to attend faculty development and maintain a community with other educators grew with increasing participation, collaboration, and knowledge acquisition (Table 4).

**Table 4:** Value of Community Representative Quotes USU: Uniformed Services University of the Health Sciences

Value of Community Themes	Representative Quotes
Shared language	I like that it gives us all a common language so that we, as the educators in a facility, can then ping things off each other, and when we go to multiple facilities, you have the same sort of environment at each place. I like how USU has kind of leveraged that across the (organization) (focus group 7).
Building commonality with others	…There’s just been kind of this progressive interest in faculty development and people talking about it more; as staff turnover, new staff come in, and they’re interested, and it’s almost kind of bringing them into a community to some extent of, okay, we do this thing together (focus group 8).
Increased collaboration	My immediate group of colleagues here within primary care is (asking), how could we incorporate more adult learning theory into that or push towards more active learning activities versus straight forward lecture? And I think I’ve seen a lot more openness but also working together to brainstorm. Oh, what would be the best way to present and share this material? (focus group 8).

Impact of leadership

The impact of leadership was the final overarching theme from the focus groups with specific subthemes of mentoring junior faculty and the impact of executive hospital leadership. The impact of leadership refers to the influence of both institutional and individual leaders on faculty development culture, access, and participation. This theme encompasses how executive support, or the lack thereof, shapes faculty motivation, mentorship practices, and the institutional prioritization of educational growth. It also highlights the critical role of visible, consistent outreach from faculty development leaders in fostering buy-in across the organization. At an individual level, faculty participants described an increased confidence and motivation to serve as mentors to junior faculty. In addition to providing guidance on professional career advancement, faculty reported new intentional discussions on academic progression during meetings with junior mentees. Faculty also described a perceived evolution over time in how higher-level hospital leaders valued and supported faculty development. Several participants noted a gradual shift at some sites, from an exclusive focus on clinical productivity to a more balanced recognition of educational contributions. This emerging cultural change was most evident where leadership actively promoted faculty development by advertising opportunities, reducing time-related barriers, and offering incentives. However, this shift was not uniformly observed across all locations. Some participants continued to report limited support, particularly in settings where leadership maintained a strong emphasis on clinical demands. While a few participants suggested that larger sites may be more likely to reflect this growing support for faculty development, the sample size was insufficient to draw firm conclusions. Nevertheless, the data suggest a trajectory of increasing institutional endorsement of faculty development in certain contexts, indicating early signs of a broader cultural shift rather than a static moment in time. The participants discussed among one another the impact of leadership in the context of disparate sites across the MHS. Faculty recognized and listed the benefits of the intentional outreach of the USU FD Program leaders. The regularly scheduled (annually to every other year) USU hospital visits played a crucial role in securing support for FD from hospital and department leaders. Recognized as valuable, these workshops have been and remain essential in overcoming FD obstacles at some healthcare institutions (Table 5).

**Table 5:** Importance of Leadership Representative Quotes USU/USUHS, Uniformed Services University of the Health Sciences; MHS, Military Health System

Importance of Leadership Themes	Representative Quotes
Building/mentoring and developing of junior faculty	I think that’s kind of the next level of education…how to be there not only for the residents but for other faculty members in nurturing those relationships as a key part of educating medicine not just being an educator of medicine (focus group 7).
Executive hospital leadership	What I would say if the most important thing is the support of the leadership group…the leadership supports canceling clinic, encouraging multiple specialties to attend, and in brief says, “Hey, you are here; you are a teacher. You are part of the teaching faculty”; it changes the mindset and perception (focus group 2).
Outreach	USU has a very good reputation, at least from where I have been across the MHS. So, getting permission to do any kind of faculty development with USUHS has been very easy because of that…. I think part of it just the appreciation for USUHS and its contribution to the mission (focus group 3).

## Discussion

We report on faculty experiences of a geographically expansive, multilocation, single-institution FD program for over a decade, specifically identifying the effects on the culture described as lived experiences of the faculty within the organizational culture. We were particularly interested in the potential impact of our FD program on organizational culture change, as our faculty are located in multiple different states and time zones while still being in the same organization. The four themes generated were the professionalization of the clinician educator role, academic legitimization, the value of community, and the impact of leadership. Each theme can be aligned with the three levels of culture in Schein’s framework [17].

Our first theme, the professionalization of the role of clinician educator, encompassed skill building and language. Using Schein’s framework, the structured FD programs with standard educational language and visible educator communities represent the initial level. The growing institutional recognition of teaching as a meaningful academic contribution described in the focus groups reflects level 2, espoused values, indicating a conscious shift in what the institution publicly values. Critically, at the deepest level of underlying assumptions, the role of education was increasingly viewed as essential to clinical care and leadership, rather than secondary to productivity by hospital executive leaders, as demonstrated by placing academic leaders on key committees. This progression through Schein’s levels reveals that the FD program did not just impart individual teaching skills; it systematically fostered a deeper cultural shift. By establishing a common language and visible community, the program laid the groundwork for the institution to explicitly value education. More significantly, the elevation of academic leaders to key decision-making bodies signifies a fundamental change in the institution’s core beliefs about the importance of education, embedding it within its leadership structure and long-term priorities. In this way, the professionalization fostered by FD not only transformed individual faculty members but also served as a powerful mechanism for broader organizational change, signaling a cultural movement toward valuing education as a core institutional priority, thereby demonstrating the potential of targeted FD initiatives to drive profound and lasting cultural transformation within a medical institution.

Academic legitimization, the second theme, described the perceived importance of academic work in the organization. A specific artifact (Schein level 1 of culture) example of this theme was the certificates achieved through the FD program, recognizing the commitment and intentional work faculty invested in their own development and to the organization. Multiple comments cited how this was a tangible goal to achieve through participation in the program. The fact that faculty actively sought and valued these certificates as markers of their development demonstrates the program’s success in establishing educational pursuits as a valued achievement within the institution. Furthermore, the recognition that these certificates may be valued outside of our organization further bolsters external legitimization as an educator, indicating a growing professional identity rooted in educational expertise. Additionally, the observation that faculty were increasingly being valued as educators by other professions and specialties, demonstrated by being asked for their opinions on programs where previously their input was not sought, signifies a shift in espoused values. This evolution, from tangible artifacts of achievement to increased interprofessional respect, illustrates how faculty development can elevate the perceived importance of educational work within an organization. By providing formal recognition and fostering expertise, the FD program not only motivated individual faculty but also subtly reshaped the organizational perception of educators, positioning them as valuable contributors beyond their specific disciplines. This highlights how targeted FD can strategically enhance the academic standing of educators within the broader institutional landscape, fostering a culture that values and seeks out educational expertise.

Our third theme was the value of community, which is often described in FD literature [24-28]. The community described specifically referred to a broader educator community where the boundaries were beyond a department and even a specific hospital. Level 1 artifacts described included the USU FD-specific site visits and the reliability of outreach from the university, both through travel and through scheduled synchronous virtual sessions. These tangible connections facilitated the initial formation of a network that transcended typical institutional divisions. Level 2 changes included the evolution of shared values among a group of educators, which actively broke down the traditional department or hospital silos. The fact that faculty members began sharing work products and even collaborated on scholarly work demonstrates a significant shift in espoused values toward openness and collective contribution. This development of a cross-institutional community fostered by the FD program reveals a key mechanism through which culture can be changed: the creation of new social networks. By providing structural support for interaction (artifacts) and nurturing shared values of collaboration, the FD program facilitated the emergence of a powerful peer network. This network, extending beyond traditional hierarchies, fostered a sense of shared identity and purpose among educators, demonstrating how FD can intentionally cultivate communities of practice that challenge existing organizational boundaries and promote a more unified, education-focused culture.

Our final theme was the impact of leadership. Unlike other themes, there were no significant level 1 artifacts directly related to leadership’s role in the FD program itself. However, faculty described a notable shift in being valued by leaders of the hospital (level 2 espoused value). The very act of leaders attending FD sessions to observe faculty receive teaching awards from the university showed a growing public acknowledgment of the importance of teaching within the institution’s stated values. More significantly, this theme demonstrated some true level 3 changes, aligning with Schein’s description of how the allocation of time reflects underlying assumptions. In our organization, the hospital executive staff actively allowed and encouraged creative scheduling for clinicians to attend FD during our FD visits. This tangible commitment of leadership resources, specifically the flexibility in time allocation, reveals a critical pathway for faculty development to influence organizational culture. By prioritizing participation in FD through scheduling accommodations, executive leadership signaled a fundamental underlying belief in the value of faculty development and, by extension, the importance of education itself. This demonstrates that leadership buy-in and active support, translated into concrete actions such as time allocation, are essential for embedding the values and outcomes of faculty development into the very fabric of the institution’s operational norms and cultural assumptions, ultimately solidifying a culture that prioritizes the development of its educators.

The organizational changes demonstrated in this study extend our understanding beyond existing literature on FD programs, which often describe more limited, cohort-based models [7,10-12,14,16,27,28]. The unique modular workshop format and the diverse participation of educators fostered a larger and more interconnected community than typically observed in traditional FD programs. While the precise threshold of faculty engagement required for widespread cultural transformation warrants further investigation, our findings suggest the considerable potential of this inclusive model to achieve broad impact within complex academic environments. Furthermore, our methodological choice of focus groups provided a valuable lens into collective experiences of culture change within this dispersed medical education system, a common structure in many institutions utilizing multiple teaching sites. This approach allowed the participants to build upon each other’s experiences, potentially surfacing nuances relevant across distinct institutional microcultures. The strength of our findings is further supported by the representation of the participants from three-quarters of our teaching hospitals and across various specialties and professions. The consistent emergence of four key themes across these diverse groups and locations underscores a broadly identified development of culture among clinical educators who engaged with the workshops, despite the variety of FD presenters. Finally, the diverse composition of our study team, encompassing multiple perspectives during the coding and thematic development process, enhances the rigor and robustness of our interpretations. Consequently, our findings hold practical implications for understanding and facilitating cultural change across similarly distributed campuses (Table 6).

**Table 6:** Practical Application for Faculty Development (FD) and Organizational Change

Theme	Practical Application for Faculty Development and Organizational Change
Professionalization of the clinician educator role	Strategically design FD programs to move beyond individual skill development to systematically foster a deeper cultural shift by establishing a common language, building visible communities, and ultimately influencing the institution’s core beliefs and leadership priorities regarding the value of education.
Academic legitimization	Design FD programs that provide formal recognition (e.g., certificates) to elevate the perceived importance of educational work. This not only motivates individuals but also enhances the academic standing of educators within the broader institution, fostering a culture that values and seeks out educational expertise across disciplines.
Value of community	Intentionally cultivate cross-institutional communities of practice through FD programs by providing structural support for interaction and nurturing shared values of collaboration. This can challenge existing organizational boundaries and promote a more unified, education-focused culture by fostering a sense of shared identity and purpose among educators.
Impact of leadership	Secure leadership buy-in and active support for FD programs, translating this into concrete actions such as flexible time allocation for participation. This signals the institution’s underlying belief in the value of faculty development and education, embedding these values into operational norms and solidifying a culture that prioritizes the development of its educators.

Limitations

One limitation is that this was only one organization. However, we have a complex organization with several suborganizations, and the themes were consistent. Next, our participants may have conflated the organization of the military with the organization of academics. This conflation is difficult to minimize in our organization’s context; however, we attempted to mitigate this by repeatedly emphasizing the distinction during the focus groups and in the invitation. Lastly, we could have selection bias where faculty most invested in the FD program were participating. However, we had some participants with more negatively phrased comments and critiques of the program, and despite this, the overall themes remained consistent.

## Conclusions

In conclusion, our study uniquely analyzes the organizational impact of a multi-institutional, geographically dispersed FD program, demonstrating its potential to drive significant cultural change. We found that FD initiatives foster change by professionalizing the educator role, leading to a deeper institutional valuing of education embedded in its leadership. The program also achieved academic legitimization of educational work, elevating the standing of educators across disciplines. Furthermore, it cultivated a vital community that transcended institutional boundaries, fostering a unified, education-focused culture. Finally, the impact of leadership in valuing and supporting FD underscored its critical role in embedding these changes. Our findings suggest that strategically designed FD programs can indeed influence organizational culture by enhancing professional identity, building community, and gaining leadership buy-in.

## Appendices

Table 7 shows the interview guide questions and rationale.

**Table 7:** Interview Guide Questions and Rationale USU: Uniformed Services University of the Health Sciences

Questions	Rationale
1. Specifically thinking about the USU faculty development program, can you describe the evolution of the educational environment, culture, and/or climate that you work in over the past five years?	This question and subsidiary questions 1a-1f were designed to evaluate shifts in visible structures and practices of the educational environment as perceived by the focus group members. These questions assisted the research team by evoking changes in artifacts such as shifts in curriculum (1 and 1f), formal and informal leadership support for faculty development (1a and 1b), interfaculty interactions, and interdepartmental interactions (1e). Question 1d was designed to assess stability between locations, as many active-duty military physician educators move depending on the needs of their organizations, as often as every 2-3 years. Schein’s model suggests that changes in visible structures and practices, or artifacts, are a result of shifts in underlying assumptions and espoused values over time.
1a. Have there been changes in how faculty and local leadership support faculty development?	
1b. Have there been changes in how the facility views the skill of teaching?	
1c. Have there been changes in how your colleagues interact with each other about educational items?	
1d. If you moved during that time, how are the cultures alike or different?	
1e. Have there been changes in your interactions between departments in the hospital?	
1f. Have there been changes in educational innovation in your programs?	
2. During that same timeframe, USU has been working toward increasing faculty support for academic development. Can you describe changes in your own academic and professional development and skills over the past five years (not including military professional education)?	Schein’s model emphasizes the importance of individual and collective learning in shaping organizational culture. This question explores how each faculty’s individual development aligns with the broader organizational changes instituted by the faculty development program.
2a. What change do you see in your interest in educational principles?	Subsidiary questions 2a-2c are designed to tap into whether underlying assumptions and deeper beliefs about professional roles and development have shifted over time.
2b. What change do you see in your reflection on personal teaching skills?	
2c. What change do you see in your own professional identity as an educator?	
3. What changes do you see in how you discuss and collaborate on education with colleagues?	Schein’s model suggests that organizational culture is largely driven by how people communicate and collaborate. This question aims to evaluate changes in artifacts in the form of interpersonal interactions and the underlying assumptions driving these changes.
4. How has the USU faculty development program shaped your views on staying in academics (even after leaving the military)?	This question is designed to explore espoused values and underlying assumptions underlying the significant decision of whether to stay in academics and the interplay with a military career. This aims to assess the stability of focus group members’ roles as educators over time and how the faculty development program has changed or solidified this role.
